# Overview of Dynamic Bond Based Hydrogels for Reversible Adhesion Processes

**DOI:** 10.3390/gels10070442

**Published:** 2024-07-04

**Authors:** Ilaria Condò, Sara Maria Giannitelli, Daniela Lo Presti, Barbara Cortese, Ornella Ursini

**Affiliations:** 1 Department of Engineering, Università Campus Bio-Medico di Roma, Via Álvaro del Portillo 21, 00128 Rome, Italy; i.condo@unicampus.it (I.C.); d.lopresti@unicampus.it (D.L.P.); 2 Department of Science and Technology for Sustainable Development and One Health, Università Campus Bio-Medico di Roma, Via Álvaro del Portillo 21, 00128 Rome, Italy; s.giannitelli@unicampus.it; 3 Fondazione Policlinico Universitario Campus Bio-Medico, Via Álvaro del Portillo 200, 00128 Rome, Italy; 4 National Research Council—Institute of Nanotechnology (CNR-Nanotec), Università La Sapienza, c/o Edificio Fermi, Pz.le Aldo Moro 5, 00185 Rome, Italy; barbara.cortese@nanotec.cnr.it

**Keywords:** dynamic hydrogels, self-healing hydrogels, adhesive hydrogels, reversible bonds

## Abstract

Polymeric hydrogels are soft materials with a three-dimensional (3D) hydrophilic network capable of retaining and absorbing large amounts of water or biological fluids. Due to their customizable properties, these materials are extensively studied for developing matrices for 3D cell culture scaffolds, drug delivery systems, and tissue engineering. However, conventional hydrogels still exhibit many drawbacks; thus, significant efforts have been directed towards developing dynamic hydrogels that draw inspiration from organisms’ natural self-repair abilities after injury. The self-healing properties of these hydrogels are closely associated with their ability to form, break, and heal dynamic bonds in response to various stimuli. The primary objective of this review is to provide a comprehensive overview of dynamic hydrogels by examining the types of chemical bonds associated with them and the biopolymers utilized, and to elucidate the chemical nature of dynamic bonds that enable the modulation of hydrogels’ properties. While dynamic bonds ensure the self-healing behavior of hydrogels, they do not inherently confer adhesive properties. Therefore, we also highlight emerging approaches that enable dynamic hydrogels to acquire adhesive properties.

## 1. Introduction

Polymeric hydrogels are soft materials with a three-dimensional (3D) hydrophilic network, able to retain and absorb large quantities of water and/or biological fluids. Due to their enhanced biocompatibility, tunable biodegradability, and customizable mechanical properties, these materials overcome the limitations imposed by conventional alternatives such as inadequate water absorption and retention, limited customization, excessive rigidity, and poor biocompatibility [1]. Over the years, they have become prime candidates for the advancement of matrices for tissue regeneration and tissue engineering (TE), 3D cell culture scaffolds, drug delivery systems, and biosensors [2,3]. Hydrogels are typically classified by taking into consideration many factors, such as network structure, composition, dimensions, sensitivity to stimuli, physical aspects, or preparation methods [4]. A detailed overview of the design and development of biopolymer hydrogels for biomedical applications was provided by Muir et al., who reviewed this issue in terms of the chemical modification and crosslinking mechanism of both natural and synthetic polymers [5]. Improving the reliability and safety of these materials represents a significant advancement for applications in the fields of medicine and biology since these features lead to better adaptability by reducing complexity and improving outcomes [6,7].

Conventional hydrogels may lose their original integrity in time, and their network structure may be affected when damaged, limiting their lifetime and their practicability. To mimic the natural ability of organisms to undergo self-repair after an injury, commonly referred to as “self-healing”, extensive research studies have been carried out [6,8,9]. The self-healing ability is based on the cleaving and reforming of dynamic linkages (also known as. “dynamic hydrogels”), and this process depends on physical or chemical events at the molecular level. An in-depth literature analysis of the design process of these hydrogels from the point of view of the mechanism behind them and their principal applications in TE has already been described elsewhere [9]. Physical self-healing processes generally include interchain diffusion, shape-memory effects, and phase separation. Chemical processes involve the introduction of dynamic bonds [10]. Both physical and chemical events may be combined to formulate self-healing hydrogels. Physical crosslinked hydrogels utilize weak intermolecular forces which are randomly arranged at crosslinking points, so they are structurally unstable and have poor mechanical properties [11]. Dynamic hydrogels are based on the presence of dynamic covalent bonds, which impart unique mechanical properties to the material network, endowing it with the ability to form reversible yet stable bonds. Dynamic bonds can continuously rearrange, ensuring a constantly moving network.

More than often, the bonding dynamicity allows the formation of reversible bonds with the external environment, imparting adhesive properties to the material. This dynamic bonding is key to its adhesiveness, as it allows the hydrogel to repeatedly adhere to and detach from surfaces. However, the self-healing capacity of dynamic hydrogels does not necessarily imply external adhesion, as the internal dynamic processes might not always translate to surface bonding and vice versa. Thus, recent research has focused on designing and fabricating dynamic hydrogels with intrinsic adhesive properties. These advancements enhance their potential applications in fields such as wound healing, TE, cell adhesion, drug delivery systems, and wearable sensors [12].

In this review, we aim to explore adhesive materials endowed with dynamic chemical bonds, focusing on how the reversibility of these bonds influences the macroscopic properties of hydrogels and the mechanisms behind their adhesiveness. We, thus, first provide an overview of the primary types of dynamic bonds from a chemical perspective and subsequently discuss the dynamic bonds within the hydrogel network to understand their implications for the material’s intrinsic properties. To provide an innovative point of view, we focused on the chemistry behind the engineering of the hydrogel network, by analyzing how the presence of different kinds of bonds, reversible dynamic and/or static covalent, can affect the final properties. Subsequently, we assess the dynamic bonds formed between the hydrogel and external surfaces, covering various adhesion mechanisms. Lastly, we provide a brief overview of the main applications of these materials, emphasizing those that highlight their potential as promising candidates [13].

## 2. Dynamic Hydrogels: General Features

Dynamic covalent chemistry involves reversible chemical reactions conducted under thermodynamic equilibrium control, allowing covalent bonds to undergo formation, breakage, and reformation [14]. Systems founded on this chemistry can adapt to changing conditions, as their molecular components can easily assemble and disassemble in response to shifts in the chemical equilibrium. The reversibility of these chemical processes is a key strength for designing hydrogels with dynamic features and with potential applications in different fields such as TE, medical dressing, smart sensors, drug delivery systems, and bioelectronics.

**Figure 1:**
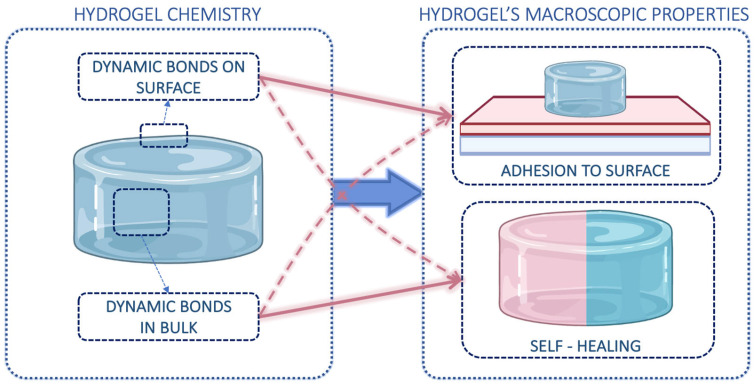
Schematic representation of the correlation between dynamic bonds and the resulting macroscopic characteristics of hydrogels. While the presence of internal dynamic bonds within the hydrogel ensures self-healing (solid line), it does not always allow adhesion to various surfaces (dashed line). Conversely, adhesion is certainly possible when hydrogels can form specific dynamic bonds with certain surfaces, albeit without guaranteeing adequate self-healing. Partially created with BioRender.com, accessed on 22 April 2023.

Incorporating reversible bonds into polymer networks yields materials with intriguing mechanical properties such as self-healing behavior, responsiveness to stimuli, and adhesiveness [15,16,17]. These bonds can break and restore in response to external stimuli, enabling dynamic covalent networks to respond to physical or chemical changes, such as variations in pH or temperature [18]. Self-healing and adhesiveness are both governed by these reversible dynamic bonds, but they are not always correlated (Figure 1). Self-healing refers to the ability of the hydrogels to repair themselves over time after damage, relying on polymeric chains and dynamic bonds present within the networks [19]. Adhesiveness is a surface property and involves the formation of junctions between a hydrogel and another surface, requiring the synergy of chemistry, topology, and mechanics [13,20]. Moreover, self-healing predominantly entails internal structural restoration, whereas adhesion primarily focuses on external interactions.

Common methods to assess self-healing include macroscopic tests, rheological measurements, and microscopic observations to confirm bond reformation and compare original and healed features [21]. Meanwhile, adhesive strength is measured through various tests such as peeling, tensile, tack, lap shear, and indentation tests [22]. Thus, the integration of dynamic covalent bonds within hydrogel networks, as well as between the hydrogel and the external environment, is decisive for creating materials with unique mechanical properties and adaptive behaviors, including enhanced self-healing and adhesiveness, and making them highly suitable for diverse applications.

## 3. Dynamic Bonds: A Chemical Recap

Dynamic bonds can be broadly divided into two main classes: covalent and non-covalent dynamic bonds. As mentioned above, dynamic covalent bonds represent a special type of chemical bond that can break and reform reversibly under certain conditions. Unlike standard covalent bonds, dynamic ones can undergo reversible exchange reactions without the need for external energy sources [14]. This property allows them to participate in self-healing and adaptive processes. They are distinct from physical interactions, such as hydrogen bonds (H-bonds) or van der Waals forces, in which the formation and breaking of actual chemical bonds, rather than purely intermolecular interactions, are involved.

A general description of dynamic bonds in polymer networks was recently reviewed in [15], where the timescales of dynamic bonds in polymeric materials were modulated and the bond exchange kinetics were correlated with the dynamic characteristics in polymer networks. In this section, we aim to recapitulate the main characteristics of each class of bonds from a purely chemical point of view to enhance the understanding of the subsequent discussion.

### 3.1. Dynamic Covalent Bonds

**Figure 2:**
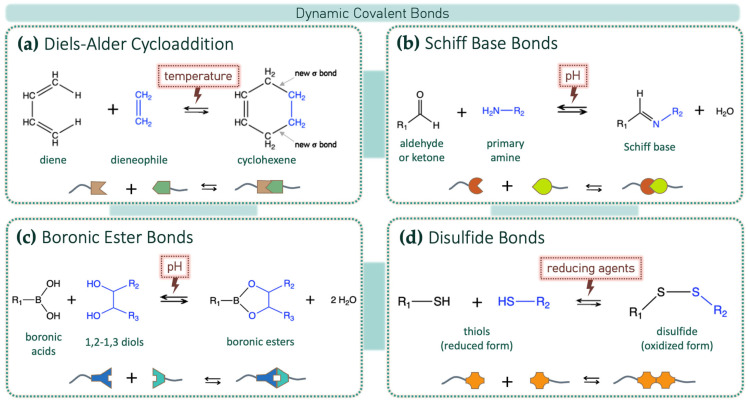
Reversible reactions of main dynamic covalent bond formation as (**a**) Diels-Alder cycloaddition, (**b**) Schiff base bonds, (**c**) boronic ester bonds and (**d**) disulfide bonds. These linkages can break and reform in response to specific stimuli that induce reversibility of covalent dynamic bonds. Changes in pH or temperature and the presence of reducing agents or competing molecules (e.g., saccharides for boronic esters) can bring back the illustrated reactions.

Covalent dynamic bonds are based on different classes of chemical reactions that are briefly described as follows in this section. The principal reactions are both chemically and schematically represented in Figure 2, indicating the specific stimuli to which they are responsive.

#### 3.1.1. Diels–Alder Cycloaddition

The Diels–Alder click reaction, reported in Figure 2a, is a thermo-reversible cycloaddition that generates stereoselective cyclic compounds. This reaction is highly selective, occurring between a conjugated diene and a dienophile (a double or triple bond co-reactant), and is widely used for assembling six-membered rings. The Diels–Alder reaction requires mild conditions and proceeds with high efficiency, without side reactions or by-products. The driving force of the reaction is the formation of new σ-bonds, which are more energetically stable than π-bonds. This concerted process means that bonds break and reform simultaneously, either autonomously or with external stimuli (e.g., heat) [23].

The thermo-reversible nature of Diels–Alder reactions allows for the dissociative retro-Diels–Alder reaction at elevated temperatures, enabling the development of thermally self-healing polymers [24]. Other types of Diels–Alder reactions, such as the heteroatom Diels–Alder reaction and the intramolecular Diels–Alder reaction, expand their usage by forming six-membered heterocycles. Due to their high selectivity, high chemical yield, and lack of by-products, these reactions are crucial methods for the synthesis of dynamic covalent hydrogels [6,9].

#### 3.1.2. Schiff Bases and R-C=N Dynamic Bonds

**Figure 3:**
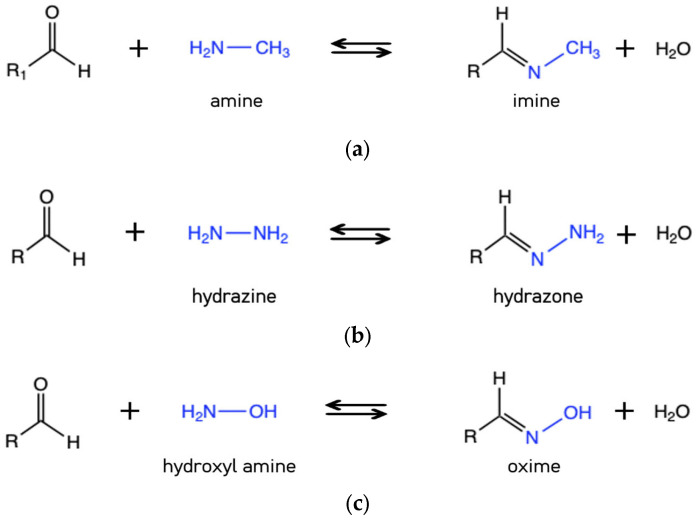
Reversible reactions for the preparation for –C=N compounds: (**a**) imine, (**b**) hydrazone, and (**c**) oxime.

The reaction between aldehydes or ketones with ammonia or primary amines forms imine derivatives, also known as Schiff bases, characterized by a C=N double bond and shown in Figure 2b. This process is reversible and can be hydrolyzed back to the corresponding amine and carbonyl or formyl compound under acidic conditions. A wide variety of substances with -NH_2_ groups can react with aldehydes or ketones through an addition–elimination sequence, yielding compounds with a carbon–nitrogen double bond. Depending on the NH_2_-substituted compound, these products are classified into imines, hydrazones, and oximes (see Figure 3).

The rate-determining step in the formation of a C=N bond is the dehydration phase [25]. The formation of Schiff bases is pH-dependent; in acidic solutions, the C=N double bond is protonated, leading to a reverse dissociation reaction. The hydrolytic stability of these compounds increases in the order imine < hydrazone < oxime, with hydrazones and oximes possessing greater intrinsic stability due to the presence of electronegative heteroatoms (N and O) in their structure [25,26].

The equilibrium of the imine formation reaction can be shifted by pH changes, resulting in the dynamic behavior of hydrogels, which exhibit reversible pH-responsive bonding as a macroscopic expression of this equilibrium shift.

#### 3.1.3. Boronic Ester Bonds

Boronic acids react with polyols in aqueous solutions to form reversible and cyclic esters. The condensation reaction between boronic acids and cis-1,2 or cis-1,3 diols, shown in Figure 2c, occurs under mild conditions at room temperature without requiring catalysts. The reversible formation of boronic esters is a promising and safe method for creating stimuli-responsive hydrogels through dynamic covalent crosslinking. Critical factors for the stability of boronic ester bonds are the binding affinity of the diol to the boronic acid and the ionization constant (pKa) of the boronic acids. The binding affinity is a measure of the strength of the interaction between the diol and the boronic acid. Following the first systematic examination of the binding affinities between boronic acids and diols [27], detailed guidelines to predict boronic ester behaviors in crosslinked polymer networks were established [28].

The stable boronate ester forms at pH values higher than the pKa of boronic acid. Boronate ester bonds dissociate under acidic conditions and reform under alkaline conditions [29]. The reactivity of boronic acid groups can be adjusted using simple neighboring group effects, resulting in materials with highly tunable mechanical properties. The behavior of these materials is directly influenced by the boronic ester structure, and through rational molecular design, the kinetics and thermodynamics of bond formation and breaking can be customized [28].

#### 3.1.4. Disulfide Bonds

A disulfide bond is a sulfur–sulfur bond typically formed from two free thiol groups (see Figure 2d). The interconversion between dithiol and disulfide groups is a redox reaction: the free thiol-bearing molecules are in a reduced state, while the disulfide form is in an oxidized state. This reaction is dynamic and reversible under physiological conditions. The ease of converting thiols to disulfides, and vice versa, endows dynamic behavior to polymers containing disulfide bonds. Disulfide bonds are cleaved to the corresponding thiol functional groups in the presence of reducing agents. The thiol–disulfide exchange reaction that occurs between thiolates and disulfides further enhances this dynamic and reversible nature, making these polymers efficient dynamic self-healers. Consequently, hydrogels crosslinked by disulfide bonds exhibit excellent self-healing properties and rapid gel formation [9].

### 3.2. Dynamic Non-Covalent Interactions

Physically crosslinked hydrogels are based on the presence of non-covalent bonds that form a network structure. The dynamic non-covalent bonds are based on rapidly exchanging supramolecular interactions, each with a characteristic timescale. In the context of dynamic bonds, the timescale of exchange is correlated with the response time of the material. Additionally, dynamic non-covalent bonds tend to exchange faster than dynamic covalent bonds under ambient conditions [15]. These interactions include H-bonding, host–guest interaction, metal–ligand coordination, π–π stacking, and electrostatic interactions. 

#### 3.2.1. H-Bonding

The H-bond is one of the simplest and most versatile non-covalent interactions. H-bond is an intermolecular force that occurs when a hydrogen atom is covalently bonded to an electronegative atom X-H and exists in the vicinity of another electronegative atom with a lone pair of electrons “Y”.

H-bonds are generally stronger than ordinary dipole–dipole bonds, but weaker than true covalent and ionic bonds. The advantages of H-bonds are the binding affinity and the dynamic modulation, which are widely tunable with the reaction parameters, such as temperature or pH, and that they can be readily created between the polar groups, linkages groups or side chains of different polymers.

#### 3.2.2. Host–Guest Interactions

A host–guest interaction is a non-covalent connection between two molecules in which a smaller guest molecule becomes encapsulated within a larger host. The host molecules usually are macrocyclic compounds such as cyclodextrin, calixarenes, and porphyrin. Host–guest systems are characterized by high levels of chemo-selectivity, particularly for recognition processes in aqueous media. The host–guest interaction is highly selective and reversible; thus, hydrogels based on this show several functionalities, such as self-healing properties, conductivity, and stimulus responsiveness (e.g., redox, pH, temperature, and light) [30].

#### 3.2.3. Metal–Ligand Coordination

The metal–ligand coordination is formed between a metal and corresponding ligands. The ligands are arranged in a simple, spatial, geometric pattern around a central atom. Hydrogels based on dynamic metal–ligand coordination show several advantages in terms of macroscopic and molecular structural stabilization as the metal–ligand coordination bonds are stable under ambient conditions and the conditions of the reversible reaction are simple [31].

#### 3.2.4. π–π Interactions

The π–π interaction is a particular type of attractive dispersion force that is established between unsaturated cyclic molecules, as molecules carrying π systems, and molecules conjugated with carbon–carbon double bonds. These interactions, in the presence of other non-covalent interactions such as H-bonds, contribute greatly to the stabilization of materials in a cooperative relationship.

## 4. Dynamic Hydrogels: Engineering the Network

An ideal hydrogel for biomedical engineering should have excellent properties, especially high stretchability, strength, resilience, and self-healing ability to withstand cyclic loading and damage [32]. As previously discussed, dynamic hydrogels are characterized by the reversibility of the bonds they exhibit, which endows them with high adaptability and self-healing capabilities [33]. Nonetheless, the reversibility of the bonds and the resulting dynamism of their networks tend to change the mechanical properties of these materials in terms of shape and stiffness [11]. Compared to permanent covalent crosslinks, reversible crosslinks provide the desired dynamic properties to hydrogels but are usually less stable under external interferences [34]. Therefore, various chemically hybrid hydrogel materials have been developed, comprising dynamic bonds for reversibility, adaptability and self-healing, covalent bonds for improved mechanical properties, and physical interactions that contribute to increasing the stability of the network.

**Figure 4:**
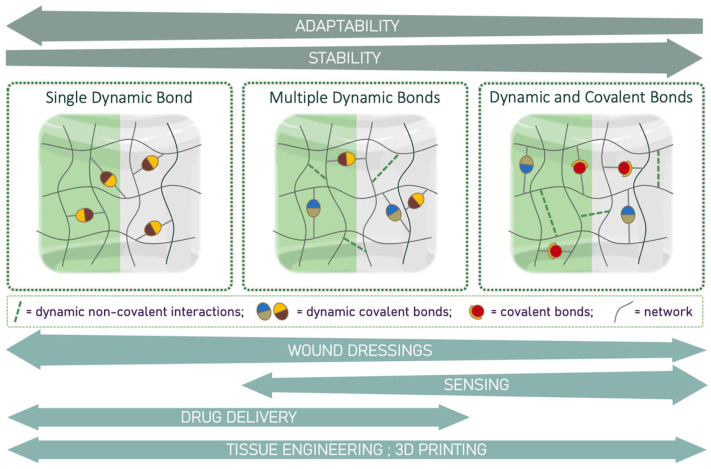
Illustration of different linkage configurations in adhesive hydrogels. Single dynamic bonds provide hydrogels with high adaptability but limited stability, making them primarily suitable for dynamic biological environments (TE), wound dressings (e.g., diabetic wound healing, post-surgical bandages, patches for tissue regeneration), drug delivery systems, and 3D printing. Hydrogels based on a combination of dynamic and covalent bonds exhibit high stability and moderate adaptability, making them ideal for sensing applications. Multiple dynamic bonds offer enhanced stability while maintaining adaptability, thus representing a compromise, allowing every mentioned application. The application arrow width is proportional to the number of works published so far.

The following section examines the current literature on dynamic hydrogels from the perspective of the network nature in terms of bonds associated with the main dynamic linkage. This analysis starts with materials featuring a singular type of dynamic bond (Section 4.1), progresses to those with multiple dynamic bonds (Section 4.2), and concludes with those incorporating both dynamic and covalent bonds (Section 4.3). A schematic representation of this section is provided in Figure 4.

### 4.1. Hydrogels Based on a Single Dynamic Bond

Dynamic hydrogels with single dynamic bonds offer promising capabilities such as high adaptability and self-healing in a very simple network, making them attractive for applications like drug delivery and TE. Their reliance on single dynamic bonds can compromise mechanical strength and long-term stability. Understanding these advantages and limitations is vital for optimizing their performance in diverse applications.

Various single dynamic bond-based hydrogels and their main polymers have been investigated in the literature and are summarized in Table 1. For example, a self-adapting hydrogel with a single Schiff base linkage was developed by Wang et al., who obtained the network using chitosan as the backbone and oxidized konjac glucomannan (OKG) as a crosslinker [35]. In this case, the easy formation of this bond ensures the production of an injectable hydrogel with self-healing properties, leading to a material adaptable to irregular wounds. Injectable hydrogels exploiting Schiff base bonds were also obtained by simply mixing non-toxic agarose (AG)–ethylenediamine conjugate (AG-NH_2_) and dialdehyde-functionalized polyethylene glycol (PEG) solutions [36]. This intrinsic linkage imparted superior tissue adhesiveness to the pH-responsive hydrogel, making it a promising candidate for long-lasting wound dressings. 

Furthermore, a multifunctional hyaluronic acid (HA)-based hydrogel incorporating Schiff base bonds has been described by Yang and colleagues and assessed for potential use in treating wounds infected by drug-resistant bacteria [37]. The network was composed of cystamine-modified HA, benzaldehyde-functionalized PEG-co-poly(glycerol sebacate), and polydopamine (PDA)-modified polypyrrole (PPy) nanocomposite. The chemical preparation of the hydrogel involved multiple Schiff base reaction sites. Initially, cystamine was grafted onto the HA framework with which the benzaldehyde-terminated PEG-co-poly(glyceryl sebacate) crosslinking agent formed Schiff base bonds. The subsequent incorporation of the PDA@PPy nanocomposites introduced more Schiff base bonding.

Hydrogels based on HA have also been developed utilizing dynamic hydrazone bonds [38,39]. For example, a disulfide-containing crosslinker, 3,3′-dithiobis(propionic hydrazide), was used to make hydrazone bonds with the aldehyde-functionalized HA to fabricate an injectable hydrogel suitable for biomedical applications [38]. The simplicity of its network makes it a good multi-responsive tissue adhesive suitable for on-demand degradation profiles under various external stimuli.

Chen’s group presented a hydrogel exploiting boronic ester bonding methodology to dynamically crosslink polyvinyl alcohol (PVA) and boric acid (BA) [41]. BA reacts with the hydroxyl groups of PVA chains, forming boronic ester crosslinks that are stable under alkaline conditions but soluble in water at neutral pH, favoring debonding. Introducing boronic ester-based dynamic covalent bonds into polymer networks enables fast and reversible adhesion and provides a promising approach for next-generation wound dressing adhesives.

Moreover, the development of dynamic hydrogels based on disulfide bonds has been described by Du et al. [43]. Benefiting from the capability of α-lipoic acid (LA) to form this dynamic bond, they directly obtained an advanced hydrogel (poly(LA-*co*-sodium lipoate) (PLAS)) through the heat- and concentration-induced ring-opening polymerization of LA in an aqueous solution. The material exhibited moderate mechanical strength, injectability, self-healing capability, and adequate adhesiveness to biological tissues with promising applications as wound dressings and skin healing by sealing injury sites. Since LA is a natural antioxidant, the resulting PLAS hydrogel also showed good biocompatibility and potent reactive oxygen species (ROS)-scavenging ability.

Considering non-covalent interactions, H-bonds in hydrogels typically act as sacrificial bonds, dissipating external energy and enhancing toughness. However, they can be easily disrupted by water molecules, reducing hydrogels’ stability and crosslinking efficiency in aqueous environments. To address this, various strategies have been explored to develop efficient H-bond crosslinking systems, such as the one reported by Yu et al. whose hydrogel based on a humic acid (HuA) and polyvinyl pyrrolidone (PVP) complex demonstrated good flexibility and self-healing ability [44]. Nevertheless, the preparation of functional hydrogels based on H-bonds that combine rapid excellent self-healing and stable mechanical properties remains an open challenge.

### 4.2. Hydrogels Based on Multiple Dynamic Bonds

In recent years, dynamic hydrogels have been engineered by simultaneously introducing multiple kinds of dynamic bonds or interactions to enhance their mechanical strength, self-healing, and environmental responsiveness. Unlike single-dynamic-bond hydrogels, which often lack robustness, these hydrogels achieve a balance between adaptability and durability [47,48]. Compared to hydrogels that combine dynamic and static covalent bonds, those with multiple dynamic bonds maintain higher flexibility and responsiveness without sacrificing structural integrity. Investigating multi-crosslinked dynamic hydrogels is a current hot topic, as shown by the increasing number of studies exceeding those on single-dynamic-bond hydrogels. Indeed, the interplay of different bonds allows for more efficient self-healing and greater resistance to deformation. Additionally, the tunability of their properties through varying bond types and ratios makes them highly suitable for a wide range of applications, providing advanced and versatile soft materials.

As shown in Table 2, the main dynamic covalent bonds present in hydrogels with multiple dynamic networks are Schiff base, boronic ester, and disulfide bonds. For example, a multitasking hydrogel with a double network based on boronate ester and disulfide bonds has been developed, leveraging their reversible and dynamic nature. The inherent heat-responsive property of poly(N-isopropyl acrylamide) (PNIPAM) and the bio-adhesion of catechol moieties resulted in a hydrogel that exhibited stimulus responsiveness and biomimetic adhesion abilities [48]. Similarly, using a one-pot rapid synthesis method, a dual-dynamic covalent crosslinked network was constructed by associating the Schiff base with borate ester bonds [49]. Here, 2-formylphenylboronic acid (2-FPBA) was used as a crosslinking agent to form Schiff base and borate ester linkages with carboxymethyl chitosan (CMCS) and epigallocatechin-3-gallate, respectively. These two bonds have also been used to obtain a self-healing hydrogel where tannic acid (TA) was firstly embedded into cellulose via Schiff base bonds and then added to PVA–borax which are linked through borate ester bonds [50]. In this case, the reversible H-bonding also contributed to the multiple dynamic and elastic 3D network, favoring the rapid self-healing properties and reliable mechanical performance of hydrogels as hemostatic wound dressings.

Indeed, a dynamic covalent bond is often accompanied by a dynamic non-covalent one (e.g., H-bonds, hydrophobic interactions, coordination bonds). Inspired by the self-assembly of natural saponin glycyrrhizic acid (GA), which crosslinks by forming H-bonds, a double-dynamic-network hydrogel was developed through a Schiff base reaction between aldehyde-containing GA and the biopolymer CMCS [51]. H-bonds also cooperated in the chemical structure of a hydrogel made of polyacrylic acid (PAA), PVA, and borax [60]. The presence of borax allowed the formation of dynamic borate ester bonds, enhancing the hydrogel’s reversible adhesion and improving the cohesion of the two main polymeric components. Being rich in carboxyl groups and hydroxyl groups improves the formation of the non-covalent bonds and the hydrogel’s adhesiveness onto various surfaces. The same couple of bonds was employed to synthetize another adhesive but also conductive hydrogel starting from sodium alginate (Alg), modified with 3-aminophenylboronic acid (3-aminoPBA) and dopamine (DA), respectively [62]. The formed dynamic boronic ester bond between 3-aminoPBA and DA endowed the hydrogel with a rapid self-healing property.

Another study presented by Liao et al. showed a PAA-based hydrogel created by polymerizing a bi-functional imidazole-type ionic liquid monomer with integrated disulfide and alkene bonds, and an octadecyl methacrylate with both disulfide bonds and hydrophobic interactions [64]. The PAA with abundant carboxyl groups gave the hydrogel good self-adhesiveness to different substrates, making it promising for various ionotronic applications. The use of dynamic disulfide bonds to construct the main backbone of the hydrogel, made more stable by multiple H-bond connections, was described in [69]. The authors fabricated a low-cost, antibacterial, and self-healable hydrogel with strong adhesion activity through natural small molecules, including thioctic acid (ThA) and gentamicin (GM). The ring-opening polymerization of ThA, led by the formation of dynamic disulfide bonds, resulted in the construction of poly(ThA). GM was introduced into the network as a physical crosslinker to increase stability through multiple H-bonds between the hydroxyl and amine groups of GM and the carboxyl groups of poly(ThA). Since the structural nature of the material allowed its degradation in water, the hydrogel could act as a continuous GM release system to achieve persistent antibacterial action.

Although these kinds of bonds create very good candidates for stimulus-responsive materials or rapid self-healable systems and offer enhanced properties with respect to single-dynamic-bond hydrogels in terms of stability, they lack of stronger mechanical properties and stability when these characteristics are required.

### 4.3. Hydrogels Based on Multi-Crosslinked Networks: Dynamic and Covalent Bonds

The combination of multiple linkers allows us to introduce complementary functionalities into a single material. Examples of multi-crosslinked hydrogels are shown in Table 3, specifying which types of bonds coexist and the main polymers involved.

Within double-network hydrogels, the first network is usually brittle, rigid, and adequately crosslinked, providing sacrificial bonds that during deformation allow the dissipation of large amounts of energy and provide the hydrogels with mechanical strength and rigidity. In contrast, the second network is often ductile, soft, and weakly crosslinked or non-crosslinked, absorbing external stress, which makes the hydrogels flexible and tough [74,75]. An example of a double-crosslinked network was reported by Yu et al., who combined the Diels–Alder click reaction with dynamic acylhydrazone bonds within the same hydrogel [76]. Here, the Diels–Alder click chemistry contributed to the hydrogel’s structural integrity and mechanical strength in physiological conditions, thus enabling application in TE and in the tissue repair field. Meanwhile, the dynamic covalent acylhydrazone bonds, being pH-responsive, endowed the hydrogel with self-healing properties and regulated the crosslink density in an on–off manner. Similarly, a hydrogel composed of Alg, PAA, and liquid metal with gallium ions was developed [77]. The performance of this hydrogel was dependent on its double network, which included physical and chemical interactions. Covalent bonding introduces a stable and strong chemical network within the hydrogel. The physical crosslink network arises from ionic interactions and H-bonds between the carboxyl groups of the PAA matrix and gallium ions. In this case, non-covalent interactions, such as H-bonding and metal coordination, also contribute to maintaining the structure but are also responsible for the hydrogel adhesiveness due to the presence of multiple functional groups (carboxyl and hydroxyl) in the Alg and PAA chains. These groups readily form H-bonds with the polar surfaces of various materials.

**Table 3:** Dynamic hydrogels based on multi-crosslinked network: dynamic, single or multiple, and covalent bonds.

Nature of Dynamic Bond	Main Polymer	Components Involved in the Main Linkage	Other Covalent and/or Dynamic Bonds *	Refs.
Schiff base bond	chitosan	HTCC-MA + OHA	**UV irradiation** HTCC-MA	[78]
		CMCS + modified PCD	**Ester bond** PVA + borax **Host–guest linkage** modified PCD	[75]
	gelatin	Gel + OKG	**Micheal addition** Gel + DA **Catechol-Quinone Coupling** DA + OKG	[79]
hydrazone bond	hyaluronic acid	HA–furan–adipic dihydrazide + HA–furan–CHO	**Diels–Alder Click Reaction** furan + imide	[76]
	sodium alginate	OSA + adipic acid dihydrazide + PEGDA	**Schiff base Bond** **UV irradiation** PEGDA	[80]
boronic ester bond	chitosan	CMCS-DA + Alg-PBA	**UV irradiation** methacrylated CMCS-DA **Schiff base Bond** CMCS + inter DA	[81]
	tannic acid	TA-Al3+ + GG-PAM-PBA	**Polymerization** Methylene bis AM **Metal Coordination** TA + Al^3+^	[82]
H-bond	tannic acid	TA + PEGDA	**Chelation** Fe + TA **Ionic interactions** Alg-NHS + Fe **UV irradiation** PEGDA	[83]
		TA + PEG	**Covalent Crosslinking** Gel + TA	[84]
	hyaluronic acid	gallol–HA + gallol–Gel	**Covalent Crosslinking** Inter-gallol moieties	[85]
metal–ligand coordination	iron (Fe)	PAA + Fe ions in silica nanoparticles	**Covalent Crosslinking** PAA + silica nanoparticles **H-bonds** PAA + Gel **Electrostatic interaction** PAA + glycerol	[86]
		GelMAC + Fe^3+^	**UV irradiation** GelMAC **Covalent Crosslinking** GelMAC + PEGDA	[87]
	gallium (Ga)	PAA + Ga	**Covalent Crosslinking** PAA + Alg	[77]
	zirconium (Zr)	PAA + Zr^4+^	**UV irradiation** PAA-MA **Enamine bonds** PAA-MA + PEI	[88]

* See Notation and Abbreviations section for abbreviations and acronyms.

Recently, there has been a notable development in hydrogels with improved mechanical behavior, coined as ‘super tough’ hydrogels, which show a compressive strength up to 350 kPa [89,90]. For such purposes, multiple network hydrogels can combine brittle and flexible networks, e.g., dynamic reversible and static irreversible bonds, to mimic the dynamicity of the ECM in tailorable polymeric systems. In this context, Aldana et al. obtained the dynamic network of their hydrogel via Schiff base condensation, forming hydrazone bonds between oxidized sodium alginate (OSA) and the adipic acid dihydrazide [80]. The static covalent network is generated through the UV irradiation of polyethylene glycol diacylate (PEGDA). The dynamic nature of reversible hydrazone bonds imparts self-healing properties, injectability, and thus 3D printability to the hydrogel, while the static network enhances its stability and toughness, making it a good candidate for biomimetic biofabrication. To better mimic the cellular microenvironment, cytocompatible printable materials that have appropriate viscosity and stability must be developed. For instance, Shin et al. generated a hydrogel ink based on HA and gelatin (Gel) (both ECM components) modified with gallol moieties, aromatic rings with three hydroxyl groups [85]. The hydrogel was formed through multiple non-covalent H-bonds between gallols and protein backbones and, in addition, through the slow and spontaneous auto-oxidation process of gallols, a covalent crosslink. 

The demand to develop biocompatible adhesives through a facile preparation method has been investigated by Li et al., who formulated a ternary complex coacervate of TA, PEG, and Gel in a simple physical blending manner [84]. In this case, a double role was played by TA since it participated in both the covalent bonds between gelatin and TA, improving the mechanical properties of coacervate, and in the dynamic non-covalent hydrogen bonds between TA and PEG, enabling the bulk matrix to dissipate energy upon deformation.

Gel was also employed by Ghovvati et al. to form a stretchable composite hydrogel consisting of gelatin methacrylol catechol (GelMAC) with ferric ions, and PEGDA [87]. The hydrogel was formed through a two-step crosslinking method, using ferric ions covalently linked through visible light. The authors hypothesized that catechol moieties of GelMAC enhanced the adhesion to the tissue while ferric ions assist in further crosslinking by chelation. This engineered material was shown to adhere to wet tissue surfaces through the chemical conjugation of catechol and methacrylate groups with the gelatin backbone.

An antifreeze conductive self-healing organogel based on a physicochemical double crosslinking network was proposed by Ge et al. [86]. The organogel was obtained via covalently linking PAA with vinyl-functionalized silicon nanoparticles to form a mechanical skeleton also equipped with a dynamic reversible crosslinking network between acrylic acid (AA) and iron ions. By the addition of the conductive polymer pPy grafted onto Gel, a balance between the physical and chemical networks was created. The organogels also adhered to different materials thanks to the reversible H-bonding provided by the hydroxyl groups of glycerol.

Finally, a self-healing, adhesive, and conductive organohydrogel was prepared through multiple dynamic interactions [88]. The random copolymer PAA–co-acetoacetoxyethyl methacrylate interacts with the branched polyethylene imine (PEI) and Zr^4+^ ions via dynamic covalent enamine bonds, coordination, and electrostatic interactions to improve stretchable, compressible, fatigue-resistant, and self-healing performance. By the addition of ionic liquids, the stability and the conductivity of the resulting gels increased.

In conclusion, the interplay between static and dynamic bonds imparts enhanced toughness, stability, and flexibility to these materials, along with excellent network adaptability, including self-repair, adhesion, and responsiveness to stimuli. This multifaceted approach opens promising avenues for the development of advanced hydrogels suitable for a wide range of biomedical and technological applications. However, optimizing the balance between different types of crosslinks and polymers remains a pressing challenge for the realization of hydrogels that achieve desired performance in specific applications.

## 5. Adhesion Mechanisms

Adhesive hydrogels are emerging as highly appealing biomedical materials due to their inherent self-adhesiveness, flexible structure, dynamic mechanical properties, and the ability to create a near-physiological environment [12,91]. However, water molecules within hydrogels do not participate in adhesion processes, and their high water content could even inhibit adhesion. Despite this challenge, hydrogels can be engineered to optimize adhesive performance through careful design. 

Hydrogel adhesion is a crucial and rapidly evolving field of research, as the integrated adhesion between hydrogel interfaces and surrounding tissues is vital for ensuring overall robustness and reliability in various biomedical applications. This adhesion is achieved through a complex supramolecular interplay involving chemical, topological, and mechanical synergies [20]. This interplay determines the macroscopic adhesive properties of hydrogels, which are directly related to junction bonds at the molecular level.

The key aspects of molecular design for engineering hydrogel adhesion have been presented by Bovone et al., who described how tailoring the junction properties can optimize adhesive performance [13]. The design of adhesion junctions is a strategic feature to link the binding chemistry to the adhesive properties of soft and hydrated interfaces. The fundamental mechanisms of tissue adhesion and rational guidelines for designing adhesives for specific tissues have been described by Nam and Mooney in [92].

In this section, we explored the chemical principles that enable dynamic hydrogels to function as adhesive hydrogels. As mentioned above, while dynamic bonds within the hydrogel network can ensure self-healing properties, they do not inherently provide adhesion capabilities. Specific chemical modifications or network bonds incorporated into dynamic hydrogels can impart these adhesive properties.

**Figure 5:**
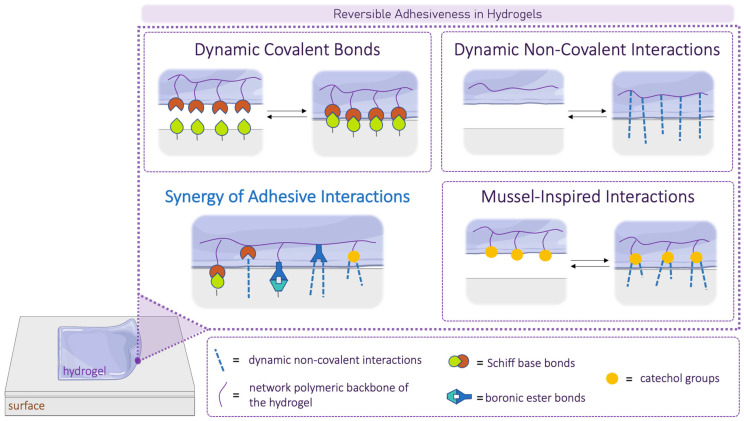
A schematic overview of main chemical adhesion mechanisms involved in dynamic hydrogel–surface interfaces. Each box illustrates representative examples of relevant adhesion bonds (e.g., Schiff base dynamic covalent bonds, between amino groups in tissues and carbonyl groups in hydrogels). Partially created with BioRender.com, accessed on 22 April 2023.

To illustrate the mechanisms by which dynamic hydrogels can also become adhesive, we have outlined the main strategies in Figure 5. We focused on four key classes of chemical interactions that confer adhesiveness to dynamic hydrogels: dynamic covalent bonds, dynamic non-covalent interactions, mussel-inspired adhesion, and the synergy of these approaches.

### 5.1. Dynamic Covalent Bonds Acting as Adhesive Bonds

Although the primary interactions involved in adhesive junctions are of physical or dynamic non-covalent nature, in this section, we present some examples of dynamic covalent bonds that take part in the adhesive mechanism.

To ensure the adhesive properties of dynamic hydrogels, the Schiff base reaction involves external chemical groups of the hydrogel and the surface tissue groups. For example, in the double-crosslinked network described in [76], the adhesive property is attributed to the Schiff base reaction between the aldehyde groups of the hydrogels and the amine groups of the cartilage tissue. 

Hydrazone bonds lead to a more stable yet still reversible binding formation. A granular HA-based hydrogel obtained through dynamic covalent inter-particle crosslinking, due to the formation of hydrazone bonds between aldehyde- and hydrazine-modified microgels, has been recently reported [39]. The adhesive properties were introduced using microgel particles, produced through extrusion fragmentation. Aldehyde- and hydrazide-containing microgels were thus mixed and jammed to form adhesive granular hydrogels that showed enhanced mechanical integrity and shape stability, allowing for stable 3D-printed structures without further post-processing. 

Incorporating an adhesive peptide into a hydrogel network is a strategy used to introduce adhesive moieties on a hydrogel surface [80]. In this case, the incorporation of aminooxy-modified Arg-Gly-Asp (RGD) peptides occurs via oxime ligation with the aldehyde groups of OSA, providing this dynamic hydrogel based on OSA and PEGDA with adhesion properties.

Boronic ester bonds, besides contributing to network construction, are often involved in conferring adhesiveness to hydrogels [41,60,82]. Recently, a PAA/PVA–borax hydrogel was proposed by introducing a double network for enhancing cohesive strength, and numerous carboxyl and hydroxyl groups, as well as borax, facilitated the hydrogen and dynamic borate bond formation, ensuring reversible adhesive capability [60]. Benefitting from the nature of these bonds, good reversible adhesion to diverse surfaces, for about 50 cycles without apparently decreasing the adhesive force, was achieved.

A hydrogel where adhesion is regulated by the potential formation and reduction of the disulfide bond at the hydrogel-modified interface was described in protein-based double-network hydrogels that mainly consisted of fibrous proteins and globular proteins [93]. To improve the usually weak mechanical properties of protein-based hydrogels, the authors developed a tough adhesion double network with bovine serum albumin and polyacrylamide (PAM). Bovine serum albumin is a typical globulin containing many functional groups, including amino, carboxyl, guanidino, and disulfide bonds, and free sulfhydryl groups, which can robustly adhere to various interfaces through disulfide bonds.

### 5.2. Dynamic Non-Covalent Interactions Acting as Adhesive Bonds

Despite the adhesion resulting from non-covalent interactions not being as strong as that from covalent bonds, in this section, we explore the role of dynamic non-covalent interactions as adhesive bonds, elucidating their versatile and responsive nature in facilitating adhesion within various materials and biological systems [92].

H-bonds are the most representative non-covalent interactions, characterized by dynamic properties due to their reversible bonding nature and broadly tunable binding affinities. Deng et al. provided an overview of H-bonding in supramolecular materials and the principles for designing H-bond-based dynamic materials [94]. When utilized as adhesive sites, hydrogen bonds offer reversible bonding with tissues. The presence of various chemical groups on tissue surfaces, such as hydroxyl, carboxylic acid, and primary amines, facilitates the formation of H-bonds with other chemical groups present in hydrogels. While a single H-bond is relatively weak, significant adhesive strength can be achieved through the presence of multiple bonds. For instance, in AA-based adhesives, repeating carboxylic groups create a high density of hydrogen bonds with tissues, enhancing adhesion [95]. However, since hydrogen bonds can be easily disrupted by water, the maintenance of adhesion often requires removing interfacial water from the tissue surface. 

H-bonding has been shown to play a crucial role in providing adhesiveness to a conductive polymeric hydrogel composed of amylopectin (Amy)/PAM–PAA [63]. The dendritic hydroxyl structure of Amy interacts with various material substrates, improving interfacial adhesion. Here, multiple H-bonds formed between the hydroxyl (-OH) groups of Amy and the carbonyl (C=O) groups of PAA with -NH_2_ and -OH groups on different substrates. Additionally, the -OH and -NH_2_ groups in Amy/PAM–PAA hydrogels enabled metal coordination with ions on material surfaces.

The adhesion mechanism of hydrogels to solid interfaces involves hydrogen bonding, hydrophobic interactions, and metal complexation, as seen in thermoresponsive dynamic hydrogels based on covalent oxime bonds [66]. The high-density H-bonds at the interface between double-network PAA/PVA–borax hydrogels and hydrophilic substrates ensure robust adhesion properties [60]. This hydrogel also showed a good adhesion to common metals due to metal coordination.

H-bonds have been shown to provide moderate adhesive properties in α-LA-based hydrogels [43]. The presence of COO− and COOH groups in this injectable hydrogel enabled moderate adhesive strength toward various substrates through the formation of hydrogen and ionic bonds.

Additionally, cooperation between multiple hydrogen bonds and dynamic disulfide bonds confers strong tissue adhesion to hydrogels constructed by crosslinking GM and ThA macromolecules [69]. The interactions between the amino and hydroxyl groups of GM and the carboxylic group side chains of poly(ThA) further enhance the adhesion properties of the hydrogel.

In summary, while adhesion resulting from non-covalent interactions may not match the strength of covalent bonds, the versatile and responsive nature of dynamic non-covalent interactions facilitates adhesion across various materials and biological systems. This type of adhesion is highly reversible, meaning it can be easily broken, and while it offers versatility, it often lacks the robustness necessary for long-lasting adhesion in biomedical conditions.

### 5.3. Mussel-Inspired Adhesion

**Figure 6:**
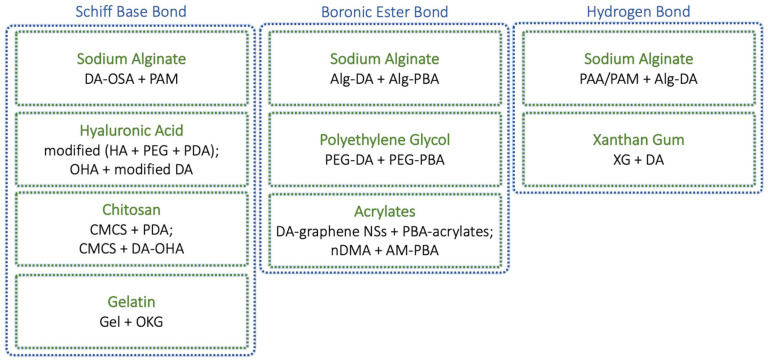
A representative schematic of dynamic hydrogels utilizing dopamine as an adhesive molecule. This block diagram presents the most relevant examples, organized by type of dynamic bond. Here, bonds are employed both in the construction of the specific network and in adhesion to external surfaces. For each bond type, we have listed the main polymers involved, followed by specific polymers and chemical groups engaged in every reported example. References: Schiff base bond (Alg [58], HA [37,57], chitosan [52,53], Gel [79]); boronic ester bond (Alg [62], PEG [42], acrylates [40,59]); H-bond (Alg [72], XG [45]). See the Notation and Abbreviations section for abbreviations and acronyms.

In recent years, the adhesion phenomenon of marine mussels, known for their strong moisture-resistant adhesion, has become a significant inspiration for the biomimetic design of hydrogel adhesives [22,96]. Engineered hydrogels aim to mimic these natural adhesive processes, which enhance their potential for biomedical applications due to their biocompatibility, biodegradability, and strong adhesive capabilities, also in wet environments (e.g., body fluids, blood, etc.). This kind of adhesion primarily benefits from mussel foot proteins (mfps), which are rich in polyphenols and key to their strong adhesive properties [97]. The main component of mfps is a catecholic amino acid called 3,4-dihydroxyphenylalanine (Dopa), a natural isomer of the immediate precursor of DA, whose presence is crucial for the wet adhesion mechanisms, promoting adhesion to surfaces through H-bonding, metal chelation, and π-π and/or cation–π interactions [98]. This has led to the proposal of a universal strategy known as the interfacial molecular lock, a strategy that involves organic and low-molecular-weight molecules containing catechol groups that can easily penetrate hydrogel networks [99]. The principle of the interfacial molecular lock relies on the combination of dynamic covalent bonds, coordination bonds, H-bonds, π-π interactions, and cation–π interactions. Furthermore, DA is often used to form dynamic adhesive hydrogels based on polysaccharides of different chemical natures. This approach leverages the versatile adhesive properties of catechol-based systems to create hydrogels with enhanced adhesion suitable for various applications [97,100,101]. In Figure 6, we present a scheme of dynamic bonds involved in mussel-inspired hydrogels where DA is employed to confer adhesive properties.

To exploit the advantages of this mechanism and enhance the properties of mussel-inspired hydrogels, DA is often grafted onto other important polymers [40,42,52,79]. For instance, Rao et al. developed multifunctional and multi-crosslinked hydrogels composed of CMCS-PDA and PAM for wound dressing applications. The interaction between CMCS and PDA is primarily based on dynamic Schiff base crosslinking since the amino groups in CMCS chains react with aldehyde functional groups. PAM provides a covalently crosslinked secondary network that advances mechanical properties (showing a compression strength that reaches 0.25 MPa when PAM is favored with respect to DA during the polymerization process) [53].

Another multi-crosslinked adhesive hydrogel based on CMCS was constructed, introducing DA to the network. In this case, to enhance tissue adhesion, DA was grafted onto the HA backbone via a Schiff base reaction, resulting in a DA-functionalized OHA, which was then incorporated into the hydrogel structure [37]. Similarly, Yang et al. developed an injectable double-network hydrogel with outstanding adhesive and self-healing properties able to accelerate full-thickness skin wound healing. DA-functionalized OHA was employed as a polymer backbone, together with adipic acid and Pluronic F127. They underwent in situ crosslinking using Schiff base dynamic covalent bonds, H-bonding, and π–π stacking interactions. The unique multi-crosslinked structure endows the hydrogel with both improved injection abilities and mechanical performance correlated with fast self-healing. OHA-DA endows the hydrogel with robust adhesion properties [57].

Additionally, Alg hydrogels based on multiple dynamic bonds using DA as adhesive molecules have been reported [58,62,72]. For example, a self-healing hydrogel was synthesized through H-bonds and dynamic Schiff base crosslinking between DA-grafted OSA and PAM chains [58]. While the covalent crosslinking provides a stable mechanical structure and the combination of physical and chemical crosslinking contributes to the hydrogel’s efficient self-healing ability, the catechol groups of DA impart tissue adhesiveness. DA served a dual function in a mussel-inspired antibacterial hydrogel with cell affinity and adhesiveness. It was developed using Alg-DA and aluminum ions to crosslink with copolymer chains of acrylamide (AM) and AA via triple dynamic non-covalent interactions, including H-bonding, coordination, and electrostatic interaction [72].

Furthermore, to mimic the adherence of marine animals to wet tissue surfaces, Huang et al. synthesized a drug-free immunogenic hydrogel using DA-conjugated xanthan gum (XG) that enabled enhanced recovery following surgical anastomosis [45].

As mentioned, mfps owe their adherence to their rich polyphenol content; thus, various hydrogels have recently been synthesized using TA, a plant-derived natural polyphenol with several catechol and pyrogallol groups in its structure [84,102]. These groups can provide multiple bonding sites capable of forming reversible covalent bonds or strong interactions with wet tissues, as H-bonds, ionic bonds, coordinate bonds, and hydrophobic interactions, thus being responsible for the adhesion mechanism. Moreover, TA possesses valuable properties such as antioxidant, antibacterial, and biodegradable capabilities, making it an ideal molecule for developing good adhesive hydrogels. For these reasons, as suggested by a literature overview, TA has attracted a great deal of interest in recent years. For example, it was used to impart adhesiveness to PVA–borax cellulose hydrogels based on reversible borate ester bonds [50]. Incorporating TA-modified bacterial cellulose (TA@BC) into the hydrogel enhanced adhesion to different substrates. In addition to its adhesive properties, TA has facilitated a catalytic mechanism with metal ions within the double-network hydrogel based on galactomannan (GG) polysaccharides. This interaction results in the formation of a conductive hydrogel that exhibits both high mechanical strength and reversible adhesion [82]. This, with both dynamic borate ester bonds and covalent crosslinking, utilizes an autocatalytic mechanism between TA and Al^3+^ ions, enabling rapid self-polymerization at ambient temperature. Furthermore, TA-modified hemicellulose (HC) nanoparticles served as nanofillers in ionic PAA-TA@HC-Al^3+^ hydrogels [67]. These hydrogels integrated covalent PAA bonds with multiple non-covalent coordination bridges. The TA@HC nanofillers, rich in catechol groups, not only provided adhesive properties but also enhanced strength, acted as dynamic link bridges, and imparted self-healing properties to the ionic hydrogel. The interaction between TA and metal ions has also been exploited in a hydrogel combining dynamic covalent bonds and supramolecular chemistry, whose network resulted from a mix of Gel–nanopolysaccharide and trivalent metal ions [56]. Here, TA provides strong cohesiveness within the material and strong adhesiveness to different substrates due to its pyrogallol/catechol groups and dendritic structures. Moreover, to introduce tunable mechanical properties and robust tissue adhesion, a multi-crosslinked hydrogel presenting various modes of covalent and non-covalent interactions was synthesized [83]. By combining and tuning different molecular interactions and crosslinking mechanisms, researchers have designed an extremely elastic and tough multifunctional hydrogel based on Alg, PEGDA, and TA interacting with Fe^3+^ ions, capable of deforming to support physiological tissue function over time under wet conditions. To emphasize the significant interest shown in TA, we report a work by Zhao et al., who made a composite hydrogel film based on carboxymethyl cellulose (CMC) with PVA/PEI and TA and proposed it for food packaging and preservation [68]. The dynamic reversible non-covalent bonds within the 3D network structures serve as sacrificial bonds. The authors observed that the adhesive strength of the film increases with the TA doping amount, attributed to the increased number of catechol groups associated with adhesion sites in the hydrogel.

The main advantage of the mussel-inspired adhesion strategy lies in its ability to maintain strong bonding even in wet environments, such as those found in biomedical contexts. Thus, exploiting dynamic interactions such as hydrogen bonding and metal chelation, along with compounds such as DA and TA, these hydrogels offer strong and versatile adhesion. Their ability to mimic natural adhesive processes while maintaining strength underscores their potential to address future challenges in TE and medical device design.

### 5.4. Synergy of Adhesive Interactions

As previously concluded concerning internal bonds within dynamic self-healing hydrogels, optimal performance in terms of strength, stability, and on-demand detachability in adhesion is achieved through a synergistic combination of various bonds [92,103]. Dynamic hydrogels exhibiting this form of adhesion have been developed from polymers with different chemical natures.

In the dual-dynamic covalent hydrogel based on CMCS and 2-formyl-phenyl boronic acid (2-FPBA), the natural polyphenol epigallocatechin gallate was used as an adhesive molecule [49]. The abundance and concentration of phenolic groups present in epigallocatechin gallate confer adhesive properties to the hydrogel. The ability to adhere to various substrates is also due to non-covalent interactions such as electrostatic interactions, π-π stacking, H-bonding, and interactions between the phenolic groups.

Additionally, the adhesion of novel supramolecular hydrogels, composed of gelatin grafted with aniline tetramer and quaternized chitosan, is governed by the synergy of adhesive interactions [55]. Yu’s group synthesized hydrogels by crosslinking monoaldehyde β-cyclodextrin via host–guest interactions and dynamic Schiff base. The tissue adhesion of the hydrogel was achieved through the synergistic action of both interactions involving protein functional groups within the tissue. In addition, the aniline tetramer provides hydrophobic interaction enhancing the interfacial interaction between the tissue surface and the hydrogel matrix.

Gel-based hydrogels were thoroughly investigated as their adhesive strength can be attributed to two simultaneous factors: cohesion and interfacial adhesiveness. Hydrogels were fabricated by introducing different concentrations of stable tricomplex molecules assembled by protocatechualdehyde (PA) and ferric iron (PA@Fe), obtaining a series of Gel-PA@Fe hydrogels [54]. PA is a kind of naturally occurring polyphenol compound. The embedded PA@Fe served as a crosslinker to improve the mechanical and adhesive properties of hydrogels through coordination bonds (catechol–Fe) and dynamic Schiff base bonds (PA–Gel). The catechol–Fe complex increases crosslinking density, enhancing the cohesion of the hydrogel and improving the interface adhesion between the hydrogel surface and the substrate.

The combination of multiple adhesive interactions also characterizes hydrogels derived from silk fibroin [61,104,105]. The union of H-bonds and intermolecular van der Waals forces confers adhesivity to composite hydrogels constituted of methacrylated silk fibroin, methacrylate Gel, and Pluronic F127 [106]. The H-bonds were generated between methacrylated gelatin (Gel-MA) and the surface of amine-rich biological tissues. The intermolecular van der Waals forces in the methacrylated silk fibroin structure also enhanced adhesion. Lastly, the H-bonds between the free PEG segment with protein-rich substrates and the hydrophobic interactions, provided by the polypropylene glycol segment with the cell membrane, promote the adhesion properties of the hydrogel.

Moreover, the synergy between H-bonds and Schiff base bonds has ruled the adhesion mechanism of a multiple-dynamic-crosslinking hydrogel based on oxidized CMCS and PVA [75]. Firstly, the hydrophilic carboxyl groups of oxidized CMCS form H-bonds with the hydrophilic group on the skin surface, improving the physical crosslinking force of the hydrogel. Then, the poly(β-cyclodextrin) (PCD) provides aldehyde groups that react with the amino groups of proteins on the same skin surface through the Schiff base reaction. Similarly, the combined effect of multiple bonds has also been crucial in determining the adhesion of multi-crosslinking polysaccharide-based hydrogels comprising modified CMCS, modified Alg, and TA [81]. Here, adhesiveness is ensured by the presence of catechol and boron–phenyl groups in the polymers. The hydroxyl groups of the catechol and boron–phenyl groups form covalent bonds with various nucleophiles, e.g., amino or thiol groups, on the tissue through Michael addition reactions and non-covalent bonds such H-bonds. The benzene rings also interact with metal ions and through cation–π and π-π stacking interactions, and the remaining aldehyde groups in the hydrogels interrelate with the amino group on the tissue via the Schiff base reaction.

H-bonding, Schiff base, and electrostatic–hydrophobic interactions conferred adhesivity to a hydrogel made of chitosan and OKG [35]. The Schiff base interaction involves the amine groups of proteins in tissues and the aldehyde groups of the hydrogel. The electrostatic and hydrophobic interactions are related to cationic chitosan (positively charged) and the negatively charged groups existing on the cell membranes.

Considering the aforementioned and being confident that a synergistic design represents the most promising approach, the chosen method to enhance adhesion in dynamic hydrogels heavily relies on their chemical composition and the dynamic bonds employed. This highlights the importance of precise engineering to achieve desired adhesive properties across various applications. The chemical composition of the hydrogel and the type of dynamic bonds it employs are key determinants in the design of adhesion junctions. Furthermore, considerations such as binding chemistry, interfacial characteristics, mechanical attributes of the gel, and the intended application domain emerge as critical factors in the engineering of hydrogel adhesion.

## 6. Applications

Dynamic bond-based hydrogels with adhesive properties hold significant potential for various biomedical applications. This section provides an overview of the key applications of these materials.

By mimicking the natural self-repair abilities of organisms, bio-adhesives with self-healing properties are excellent candidates for tissue regeneration applications to advance natural self-healing processes [107]. This includes general tissue regeneration, often correlated with hemostasis ability, as well as medical systems that assist and guide the healing process, often referred to as wound dressings [98]. This includes their use as dressings for surgical anastomosis, as demonstrated by Huang et al. with their marine-inspired hydrogel capable of adhering to wet tissue surfaces [45].

Recently, Li et al. developed a novel dual network-based hybrid and bioactive hydrogel to promote the healing of bacterial-infected wounds. The hydrogel exhibited excellent stability and mechanical performance, along with multifunctional properties such as injectability, shape adaptation and remodeling, self-healing, and strong adhesive abilities [51]. In another study, Lu et al. fabricated a tough hydrogel based on silk fibroin with robust underwater adhesion, capable of remaining in place for six months. The evaluation of adhesion and wound closure strength on blood-covered substrates showed values 7 and 13 times higher than those of commonly used cyanoacrylate glue, respectively. The proposed hydrogel performance was further investigated in in vivo animal models, showing that it was an effective tool for fast wound closure and hemostasis [104].

Similarly, these materials are of great interest in diabetic wound healing processes due to the hydrogels’ biocompatibility and resorbable nature, which minimizes the trauma associated with the removal of conventional dressings [108]. For instance, a tissue-friendly healing hydrogel based on histidine, an essential amino acid crucial for tissue formation, was obtained. This exhibited good injectability, adhesiveness, and antibacterial properties, and significantly promoted the healing of infected diabetic wounds within less than two weeks [70]. In this scenario, Qiu et al. recently developed a dual-crosslinked hydrogel based on HA and chitosan. This hydrogel formed dynamic covalent bonds with cartilage surfaces, promoting articular regeneration [78]. Li et al. fabricated a reinforced bio-adhesive for skin wound repair using a simple physical blending method with TA, PEG, and gelatin [84]. TA provided the material with bio-adhesiveness to the skin, and the presence of covalent bonds enhanced its mechanical properties. The dynamic H-bonds between TA and PEG allowed the bulk matrix to dissipate energy during skin deformation. 

Dynamic hydrogels are also ideal as drug delivery systems since the dynamic nature of these bonds allows the disruption of the hydrogel matrix in response to various physiological stimuli, such as changes in pH, redox conditions, and temperature, thereby fine-tuning the release profile of the encapsulated drugs [18]. For example, a hydrogel prepared using an aldehyde–amine Schiff base reaction showed a pH-controlled drug release pattern, where a change in pH from 7.4 to 2.0 accelerated drug release from 60% to 90% [109]. Additionally, the adhesiveness enhances hydrogel retention at the target site, ensuring localized delivery and reducing administration frequency. These also optimize therapeutic efficacy and patient compliance while minimizing systemic side effects. Chen et al. designed novel intrinsically adhesive hydrogels incorporating a multiple dynamic hydrogen network with a unique “load sharing” effect and adhesion to a range of surfaces, including glass, plastic, wood, and biological tissues, even without any chemical reaction [110]. The tissue-adhesive bandages developed can be used for a sustained release of chemotherapeutic nanodrugs for liver cancer treatment. Furthermore, taking inspiration from natural mussels, a multiple dynamic interaction-based hydrogel with strong tissue adhesion (more than 30 kPa) was recently introduced for transdermal drug delivery [111]. The synergistic effect of multiple dynamic bonds improved cohesive strength and enhanced adhesion as well as conferring exceptional thermal stability and controlled release capabilities for the target application. 

However, there are currently limited effective applications in this field as these materials are still under deep characterization and study. Despite the few examples currently existing, this remains a promising area of exploitation.

Another context in which highly adhesive and adaptable materials can offer numerous advantages is in sensing systems, especially skin wearables. These hydrogels are often equipped with molecules or substances that impart conductivity to the material, enabling the transmission and subsequent processing of monitored signals, such as CNTs, free ions, or an ionic liquid medium [61,62,64,75,88]. For instance, a hydrogel comprising a triple polymeric network was utilized as a multifunctional layer for the successful performance of either electrophysiological cardiac/muscular on-skin sensors or as an interactive stretchable human–machine interface [112]. It comprised an irreversible amide linkage and dynamic covalent bonds, which were simultaneously capable of high stretchability, efficient strain dissipation, low electrical resistance, and even robust skin adhesiveness. Another example is provided by Zeng et al., who fabricated a hydrogel sensor based on Alg, PAM and Fe^3+^, endowed with a dynamic coordination dual network that showed super stretchability, excellent adhesion (61.8 kPa adhesion strength on glass), and conductivity, promoting its application for wearable electronic devices and intelligent monitoring systems [71].

## 7. Conclusions and Future Outlook

The current state of research on dynamic hydrogels with adhesive properties is summarized here. Although research on dynamic bond-based hydrogels has made significant progress, highlighting their potential due to their versatility, several challenges remain, especially in translating these materials from research to clinical or practical applications. While dynamic bonds provide features like self-healing and responsiveness to external stimuli, they often fall short in terms of mechanical robustness required for practical use. Thus, integrating multiple dynamic bonds or stable covalent bonds is crucial to balance adaptability and strength.

The choice of hydrogel materials should be guided by the specific application requirements. Simple dynamic bond-based hydrogels are ideal when high adaptability and self-healing are needed. Hydrogels with multiple dynamic bonds offer enhanced stability and mechanical strength, suitable for scenarios requiring robustness without sacrificing dynamic responsiveness and flexibility. Hydrogels that combine dynamic and covalent bonds strike a balance between high strength and moderate dynamicity, resulting in durable materials with self-healing capacity. 

Dynamic hydrogels are not inherently adhesive, and achieving both adhesiveness and inherent cohesion in a single material remains challenging. Conditions promoting strong cohesion (gelation) can conflict with those required for good adhesion, especially in the humid and dynamic environments of biological tissues [45,104]. Despite these challenges, the inherent dynamism of these hydrogels, characterized by their ability to form and reform bonds in response to various stimuli, still provides a unique advantage that could revolutionize their biomedical applications.

Future advancements depend on discovering new dynamic bonds with superior properties and interdisciplinary collaboration to overcome current challenges and finally turn these innovative materials into practical solutions. Large-scale production with uniform properties and reproducibility is also important for clinical applications.

In conclusion, dynamic bond-based hydrogels represent a promising frontier in biomedical materials science, with the potential to revolutionize regenerative medicine and biomedical technologies by addressing current challenges and leveraging future opportunities.

## Figures and Tables

**Table 1:** Dynamic hydrogels based on a single dynamic bond.

Nature of Dynamic Bond	Main Polymer	Components Involved in the Main Linkage *	Refs.
Schiff base bond	chitosan	chitosan + OKG	[35]
	polyethylene glycol	DF-PEG + AG-NH_2_	[36]
	hyaluronic acid	modified (HA + PEG + PDA)	[37]
hydrazone bond	hyaluronic acid	modified HA + disulfide crosslinker	[38]
		modified HA	[39]
boronic ester bond	acrylates	nDMA + AM-PBA	[40]
	polyvinyl alcohol	PVA + BA	[41]
	polyethylene glycol	PEGDA + PEG-PBA	[42]
disulfide bond	lipoic acid	α-LA + SL	[43]
H-bond	polyvinyl pyrrolidone	HuA + PVP	[44]
	xanthan gum	XG + DA	[45]
metal–ligand coordination	aluminium	PAA + Al^3+^; TA@CNCs	[46]

* See the Notation and Abbreviations section for abbreviations and acronyms.

**Table 2:** Dynamic hydrogels based on multiple dynamic bonds.

Nature of Dynamic Bond	Main Polymer	Components Involved in the Main Linkage *	Other Dynamic Bonds *	Refs.
Schiff base bond	chitosan	CMCS + 2-FPBA	**Boronic Ester bond** 2-FPBA + modified gallate	[49]
		CMCS + AGA	**H-bond** intra AGA	[51]
		CMCS + OHA	**Disulfide (-S-Ag-S-)** modified Col + Ag	[52]
	gelatin	CMCS + PDA	**H-bond** CMCS + PDA	[53]
		Gel + PA	**Metal Coordination** PA + Fe	[54]
		Gel + monoaldehyde β-cyclodextrin	**Host–guest interaction** Chitosan + monoaldehyde β-cyclodextrin	[55]
		Gel + modified cellulose	**Metal Coordination** TA + Fe	[56]
		OHA + DA@OHA; OHA + micelle	**H-bond; π-π interactions** DA catechol groups	[57]
	sodium alginate	DA grafted OSA + PAM	**H-bond** Inter DA grafted OSA	[58]
boronic ester bond	acrylates	DA@graphene NSs + acrylate-PBA	**H-bond** Inter DA@graphene NSs	[59]
	polyvinyl alcohol	PVA/TA@cellulose + borax	**H-bond** Inter PVA; Cys/TA@cellulose + PVA **Schiff base Bond** Cys + TA for TA modified cellulose	[50]
		PVA + borax	**H-bond** PAA + PVA/borax	[60]
		PVA + borax	**H-bond** TA + PVA; TA + silk fibroin	[61]
	poly(N-isopropyl acrylamide)	catechol-functionalized PNIPAM + PBA	**Disulfide Bond** inter sulfide-containing PBA	[48]
	sodium alginate	Alg-DA + Alg-PBA	**H-bond** Inter Alg; CNTs + Alg	[62]
	amylopectin	Amy + borax	**H-bond** Inter Amy; Amy + PAM; Amy + PAA	[63]
disulfide bond	imidazole-type ionic liquid monomer	ionic liquid monomer with disulfide and alkene groups	**Hydrophobic interactions** Micellization of octadecyl-MA	[64]
	poly(N-isopropyl acrylamide)	RS-Ag + crosslinker with disulfide groups	**H-bond** PNIPAM + RS-Ag **Metal Coordination** RS-Ag	[65]
oxime bond	hyaluronic acid	OHA + modified Pluronic F127	**Hydrophobic interactions** Inter-modified Pluronic F127	[66]
H-bond	tannic acid	TA@HC nanoparticles + PAA	**Metal Coordination** TA@HC nanoparticles + Al^3+^	[67]
		TA + PVA	**Coordination bond** TA + CMC **Amide bond** PEI + CMC	[68]
	thioctic acid	poly(ThA) + GM	**Disulfide Bond** Inter poly(ThA)	[69]
	sodium alginate	Histidine + Alg	**Metal Coordination** Histidine + Zn^2+^	[70]
		PAM + Alg	**Metal Coordination** Alg+ Fe^3+^	[71]
		PAA/PAM + Alg-DA	**Metal Coordination** PAA/PAM + Al^3+^ **Electrostatic interaction** PAA/PAM + Alg-DA	[72]
		Lignosulfonate + PVP	**Hydrophobic interactions** Lignosulfonate + PVP	[73]

* See Notation and Abbreviations section for abbreviations and acronyms.

## Data Availability

Data sharing is not applicable.

## Notation and Abbreviations

*A*@*B*	chemical *B* modified with chemical *A*
2-FPBA	2-formyl-phenyl boronic acid
3-aminoPBA	3-amino-phenyl boronic acid
AA	acrylic acid
AM	acrylamide
AG	agarose
AG-NH2	agarose–ethylenediamine conjugate
AGA	aldehyde-contained glycyrrhizic acid
Alg	sodium alginate
Amy	amylopectin
BA	boric acid
CMC	carboxymethyl cellulose
CMCS	carboxymethyl chitosan
CNCs	cellulose nanocrystals
CNTs	carbon nanotubes
Col	collagen
Cys	cysteine
DA	dopamine
DF-PEG	dialdehyde-functionalized polyethylene glycol
Dopa	3,4-dihydroxyphenylalanine
Gel	gelatin
GelMAC	gelatin methacrylol catechol
GG	galactomannan
GM	gentamicin
HA	hyaluronic acid
HC	hemicellulose
HTCC	N-(2-hydroxypropyl)-3-trimethylammonium chitosan chloride
HuA	humic acid
LA	lipoic acid
MA	methacrylated
mfps	mussel foot proteins
nDMA	nitro-dopamine methacrylamide
NS	nanosheets
OHA	oxidized hyaluronic acid
OKG	oxidized konjac glucomannan
OSA	oxidized sodium alginate
PA	protocatechualdehyde
PAA	polyacrylic acid
PAM	polyacrylamide
PBA	phenyl boronic acid
PCD	poly(β-cyclodextrin)
PDA	polydopamine
PEG	polyethylene glycol
PEGDA	polyethylene glycol diacrylate
PEI	polyethylene imine
PNIPAM	poly(N-isopropyl acrylamide)
PPy	polypyrrole
PVA	polyvinyl alcohol
PVP	polyvinyl pyrrolidone
SL	sodium lipoate
TA	tannic acid
ThA	thioctic acid
XG	xanthan gum
